# Trade-offs between host tolerances to different pathogens in plant–virus interactions

**DOI:** 10.1093/ve/veaa019

**Published:** 2020-03-18

**Authors:** Nuria Montes, Viji Vijayan, Israel Pagán

**Affiliations:** v1 Centro de Biotecnología y Genómica de Plantas UPM-INIA and E.T.S. Ingeniería Agronómica, Alimentaria y de Biosistemas, Universidad Politécnica de Madrid, Autopista M40, km.38, Pozuelo de Alarcón, Madrid 28223, Spain; v2 Fisiología Vegetal, Departamento Ciencias Farmacéuticas y de la Salud, Facultad de Farmacia, Universidad San Pablo-CEU universities, Boadilla del Monte, Madrid, Spain and Servicio de Reumatología, Hospital Universitario de la Princesa, Instituto de Investigación Sanitaria (IIS-IP), Madrid, Spain

**Keywords:** *Arabidopsis thaliana*, *Cucumber mosaic virus* (CMV), evolution of tolerance, resistance, tolerance-tolerance trade-offs, *Turnip mosaic virus* (TuMV)

## Abstract

Although accumulating evidence indicates that tolerance is a plant defence strategy against pathogens as widespread as resistance, how plants evolve tolerance is poorly understood. Theory predicts that hosts will evolve to maximize tolerance or resistance, but not both. Remarkably, most experimental works failed in finding this trade-off. We tested the hypothesis that the evolution of tolerance to one virus is traded-off against tolerance to others, rather than against resistance and identified the associated mechanisms. To do so, we challenged eighteen *Arabidopsis thaliana* genotypes with *Turnip mosaic virus* (TuMV) and *Cucumber mosaic virus* (CMV). We characterized plant life-history trait modifications associated with reduced effects of TuMV and CMV on plant seed production (fecundity tolerance) and life period (mortality tolerance), both measured as a norm of reaction across viral loads (range tolerance). Also, we analysed resistance-tolerance and tolerance-tolerance trade-offs. Results indicate that tolerance to TuMV is associated with changes in the length of the pre-reproductive and reproductive periods, and tolerance to CMV with resource reallocation from growth to reproduction; and that tolerance to TuMV is traded-off against tolerance to CMV in a virulence-dependent manner. Thus, this work provides novel insights on the mechanisms of plant tolerance and highlights the importance of considering the combined effect of different pathogens to understand how plant defences evolve.

## 1. Introduction

Parasitism is the lifestyle of 50 per cent of all known organisms (Poulin and Morand 2000). This means that, along their lifespan, hosts will be recurrently challenged by parasites. Parasites may be pathogens, causing diseases that have a negative impact on the fitness of infected hosts, i.e. virulence (Read 1994; Anderson *et al.* 2004). To cope with pathogens, hosts have developed a variety of defence mechanisms to avoid/limit infection and its negative effects (Agnew, Koella, and Michalakis 2000), which have relevant consequences for the fitness of both interacting partners (Woolhouse *et al.* 2002). Thus, investigating the evolution and the mechanistic basis of these defences is central to understand the dynamics of host–pathogen interactions (Jones and Dangl 2006; Pagán and García-Arenal 2018).

The two main host defences against pathogens are resistance, i.e. the host’s ability to limit pathogen multiplication (Clarke 1986; Strauss and Agrawal 1999), and tolerance, i.e. the host’s ability to reduce the effect of infection on its fitness at a given pathogen load (Little *et al.* 2010; Råberg 2014). They represent two different strategies to deal with pathogens: resistance reduces the risk of infection and the multiplication rate of the pathogen, whereas tolerance does not. Hence, it is predicted that if hosts evolve resistance the prevalence of the pathogen in the host population will decrease, whereas tolerance will have the opposite effect (Roy and Kirchner 2000). Consequently, both resistance and tolerance may have significant, but different, impact on the dynamics of host and pathogen populations (Roy and Kirchner 2000; Pagán and García-Arenal 2018). Researchers have devoted considerable effort to understand the molecular basis and evolutionary consequences of resistance to pathogens. However, tolerance has received comparatively less attention, and the processes shaping its evolution are only partially understood (Little *et al.* 2010; Pagán and García-Arenal 2018).

A body of mathematical work has modelled the conditions in which tolerance evolves. Early models assumed that resources are limited and can be diverted into resistance or tolerance, but not both, and predicted that tolerance or resistance would prevail because they were mutually exclusive (van der Meijden, Wijn, and Verkaar 1988; Herms and Mattson 1992). More recent models incorporated the idea that resistance and tolerance might not be fully exchangeable, and predicted that both defence mechanisms would co-exist, with host fitness maximized: 1, only at maximum tolerance or maximum resistance (Mauricio, Rausher, and Burdick 1997; Boots and Bowers 1999) or 2, at intermediate levels of both (Restif and Koella 2003, 2004; Fornoni *et al.* 2004). All these scenarios can accommodate a trade-off between resistance and tolerance (Best, White, and Boots 2008). However, there is remarkably little experimental support for such trade-off in host–pathogen, and particularly in plant–pathogen, interactions. Indeed, most studies on plant viruses (Carr, Murphy, and Eubanks 2006; Pagán, Alonso-Blanco, and García-Arenal 2007, 2009; Montes, Alonso-Blanco, and García-Arenal 2019), bacteria (Kover and Schaal 2002; Goss and Bergelson 2006) and fungi (Simms and Triplett 1994) failed in finding a resistance-to-tolerance negative association.

**Figure 1.**
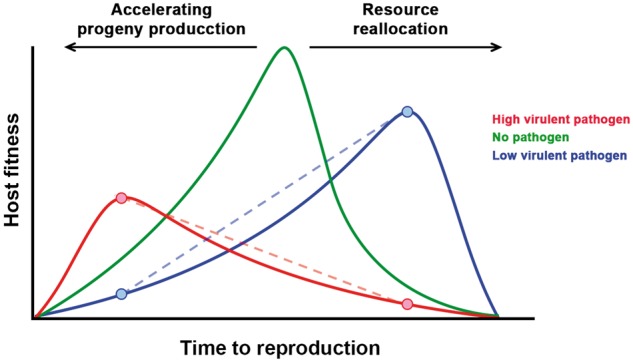
Which way to go? According to the life-history theory, hosts would modify their time to reproduction in opposite ways in order to achieve tolerance: when infected by a highly virulent pathogen (red line), hosts would bring forward reproduction to produce progeny before death; and when infected by a low virulent pathogen (blue line), host would delay reproduction so they can reallocate resourced from growth to reproduction. These strategies would maximize fitness in the presence of one virus at the cost of reducing fitness in the presence of the other (crossed dotted lines), establishing a tolerance-tolerance trade-off.

A possible explanation for this lack of support of a resistance-tolerance trade-off is that other forces might come into play in shaping the evolution of plant defences. In nature, plant populations are challenged by multiple pathogens (Syller 2012), not necessarily coinfecting the same individuals, and the evolution of tolerance to one pathogen may depend on the interaction with tolerances to others. According to the life-history theory, hosts may achieve tolerance to pathogens through modifications of their life history (Minchella 1985; Stearns 1992). In this context, the definition of tolerance is more closely related to how infected hosts reallocate resources to the different environments created by a parasite; whereas mathematical models discussed above assume that tolerance acts through a direct reduction of the parasite-induced damage by lowering virulence/increasing reproduction. However, both approaches coincide in that the final goal of tolerance is to reduce the negative effect of parasite infection on the host fitness. Life-history changes may respond to two contrasting mechanisms: Highly virulent pathogens will induce shorter host pre-reproductive, and longer reproductive, periods in order to produce progeny before resource depletion, castration or death. Conversely, less virulent pathogens will induce host resource reallocation from growth to reproduction, a delay in host reproduction or both responses at the same time, which would allow compensating the pathogen effect on host fitness (Hochberg, Michalakis, and de Meeus 1992; Gandon, Agnew, and Michalakis 2002). Hence, depending on the pathogen’s virulence, tolerance may require markedly different, even opposed, host responses that likely are difficult to maximize simultaneously. As a consequence, trade-offs between tolerances to different pathogens might be important forces for the evolution of plant defences (Fig. 1). Interestingly, such trade-offs have seldom been considered in mathematical models or in experimental analyses (Kutzer and Armitage 2016; Pagán and García-Arenal 2018).

To address this central question to understand how plant defences against pathogens evolve, we utilized *Turnip mosaic virus* (TuMV, *Potyviridae*) and *Cucumber mosaic virus* (CMV, *Bromoviridae*) and *Arabidopsis thaliana* (from here on ‘Arabidopsis’, Brassicaceae). Both viruses are commonly found in wild populations of Arabidopsis at up to 80 per cent prevalence (Pagán *et al.* 2010), indicating that the Arabidopsis–TuMV and Arabidopsis–CMV interactions are significant in nature. CMV infection moderately reduces seed production, rarely inducing sterility and has little effect on plant life period (Pagán, Alonso-Blanco, and García-Arenal 2007, 2008; Hily *et al.* 2016; Montes, Alonso-Blanco, and García-Arenal 2019). Thus, CMV can be considered as a moderately virulent virus. On the other hand, TuMV infection affects Arabidopsis flower and silique viability, which may severely affect plant fertility and often leads to sterility (Sánchez *et al.* 2015). Moreover, this virus greatly reduces plant life period (Vijayan *et al.* 2017). Therefore, TuMV can be regarded as a highly virulent pathogen in Arabidopsis, although milder TuMV genotypes exist (Sánchez *et al.* 2015). Interestingly, although both viruses have high prevalence and share common vectors (e.g. Fujisawa 1985), in Arabidopsis natural populations CMV+TuMV mixed infections occurred at low frequency and single infections are predominant (Pagán *et al.* 2010). Currently, it is not known which mechanisms determine this infection exclusion pattern. Regardless of the mechanisms involved, the coexistence of both CMV and TuMV infection phenotypes opens the possibility of evolving different tolerance responses to these two viruses that vary in virulence. Tolerance to CMV varies across Arabidopsis genotypes as a quantitative trait; and long-lived genotypes with low seed production to total biomass ratio (Group 1 genotypes) are more tolerant than short-lived genotypes that have high seed to biomass ratio (Group 2 genotypes) (Pagán, Alonso-Blanco, and García-Arenal 2008, 2009; Hily *et al.* 2016; Shuckla, Pagán, and García-Arenal 2018). This differential response has been shown to be robust across temperatures and light intensities (Hily *et al.* 2016; Montes and Pagán 2019). Tolerance to CMV in Group 1 genotypes is attained through modifications of life-history traits, mainly the reallocation of resources from growth to reproduction and, to a lesser extent, elongation of the pre-reproductive period (Pagán, Alonso-Blanco, and García-Arenal 2008; Shuckla, Pagán, and García-Arenal 2018). Virus-induced resource reallocation appears to be CMV specific, and it is not triggered upon TuMV infection (Shuckla, Pagán, and García-Arenal 2018). However, these authors used a reduced set of Arabidopsis genotypes, and did not test virulence-specific modifications of other life-history traits that would confer tolerance, and their potential trade-offs.

The key variables for measuring tolerance may vary depending on each plant–pathogen interaction (Day 2002; Rohr, Raffel, and Hall 2010). For instance, pathogens may affect plant fecundity directly or through reducing survival. In plants infected by a sterilizing pathogen such as TuMV, enhanced survival may represent the difference between reproducing or dying during the growth period. Thus, considering both the effect of infection on plant progeny production (fecundity tolerance) and survival (mortality tolerance) may be equally important to understand the evolution of tolerance. Conversely, plant mortality tolerance might be less relevant upon infection with a milder pathogen such as CMV, as infected plants generally reach the adult stage and reproduce. However, in most experimental analyses of tolerance to plant pathogens host fitness was measured only as progeny production (Pagán and García-Arenal 2018), and the relationship between fecundity tolerance and mortality tolerance have been seldom analysed (Pagán, Alonso-Blanco, and García-Arenal 2008; Shuckla, Pagán, and García-Arenal 2018). Another point under debate in the literature on plant tolerance is how it is quantified. Most often, tolerance has been measured as the effect of infection at a given pathogen load (i.e. point tolerance) (Pagán and García-Arenal 2018). At odds, it has been proposed that a more informative approach is quantifying tolerance as the slope of a regression of host fitness against pathogen load (i.e. range tolerance); the steeper the slope, the lower the tolerance, which cannot be measured on a single plant but across individuals of a given host type (e.g. genotype) (Little *et al.* 2010; Kutzer and Armitage 2016). Notably, range tolerance to plant pathogens has been seldom analysed to date (Pagán and García-Arenal 2018).

Herein, we analyse whether Arabidopsis achieves (range) tolerance to TuMV infection and if such tolerance is related to modifications of plant life-history traits. Specifically, we analysed the association between the effect of infection on plant progeny production (fecundity tolerance) and life period (mortality tolerance) with resource reallocation from growth to reproduction and with modifications in the length of the growth and reproductive periods. We also analysed resistance-tolerance trade-offs upon infection by TuMV and CMV, and if tolerance to TuMV is traded-off against tolerance to CMV.

## 2. Materials and methods

### 2.1 Viruses and Arabidopsis genotypes

Viruses UK1-TuMV (Acc.N. AB194802), JPN1-TuMV (Acc.N. KM094174) and LS-CMV (Acc.N. AF127976) were used. JPN1-TuMV was obtained from a field-infected plant of *Raphanus sativus* (Brassicaceae) and UK1-TuMV from a plant of *Brassica napus* (Brassicaceae). LS-CMV was originally obtained from a field-infected plant of *Lactuca sativa* (Asteraceae). All viruses were derived from biologically active clones (Zhang *et al*., 1994; Sánchez *et al.* 1998; López-González *et al.* 2017) by *in vitro* transcription with T7 RNA polymerase (New England Biolabs, Ipswich, USA), and transcripts were used to infect *N.benthamiana* plants for virus multiplication. We used a single CMV isolate because previous analyses indicated that, in Arabidopsis, the fraction of the variance in virulence/tolerance explained by the CMV isolate is very low (4%) (Pagán, Alonso-Blanco, and García-Arenal 2007), which is not the case for TuMV. Indeed, UK1-TuMV and JPN1-TuMV have different levels of virulence in Arabidopsis (Sánchez *et al.* 2015; Montes and Pagán 2019). This allowed exploring whether variation in tolerance to TuMV and CMV were species specific or virulence dependent.

We used ten genotypes representing the Eurasian geographic distribution of the species and eight representing its distribution in the Iberian Peninsula, a Pleistocene glacial refuge for Arabidopsis (Sharbel, Haubold, and Mitchell-Olds 2000) (Table 1). Seeds were stratified for seven days at 4°C in 15 cm-diameter pots, 0.43 l volume containing 3:1, peat: vermiculite mix. Afterwards, pots were moved for seed germination and plant growth to a greenhouse at 22°C, 16 h light (intensity: 120–150 mol s/m^2^), with 65–70 per cent relative humidity. In these conditions, plant genotypes conformed two allometric groups (Table 1 and Supplementary Fig. S1) as described previously (Pagán, Alonso-Blanco, and García-Arenal 2008). Because plant allometry has been repeatedly reported as a relevant factor to understand Arabidopsis tolerance to virus infection (Pagán and García-Arenal 2018), allometric group was considered as a factor in all analyses. Plants were mechanically inoculated, either with *N.benthamiana* TuMV- and CMV-infected tissue ground in 0.1 M Na_2_HPO_4_ + 0.5M NaH_2_PO_4_+0.02 per cent DIECA, or with inoculation buffer for mock-inoculated plants. Inoculations were done when plants were at developmental stages 1.05–1.06 (Boyes *et al.* 2001). After inoculation, all individuals were randomized in the greenhouse. For each Arabidopsis genotype, seven to ten plants per virus were inoculated, and other seven were mock inoculated.

**Table 1.** Arabidopsis genotypes used in this work, their geographical origin, and allometric group/subgroup.

Genotype	Origin	Allometric group (subgroup)
Cum-0	Cumbres Mayores (Spain)	Group 1 (a)
Kas-0	Kashmir (India)	Group 1 (a)
Ll-0	Llagostera (Spain)	Group 1 (a)
Cad-0	Candelario (Spain)	Group 1 (b)
Cdm-0	Caldas de Miravete (Spain)	Group 1 (b)
Kas-2	Kashmir (India)	Group 1 (b)
Kyo-1	Kyoto (Japan)	Group 1 (b)
An-1	Amberes (Belgium)	Group 2
Bay-0	Bayreuth (Germany)	Group 2
Col-0	Columbia (unknown)	Group 2
Cvi	Cape Verde Islands	Group 2
Fei-0	Santa María da Feira (Portugal)	Group 2
L*er*	Landsberg (Poland)	Group 2
Cen-1	Centenera (Spain)	Group 2
Mer-0	Mérida (Spain)	Group 2
Pro-0	Proaza (Spain)	Group 2
Shak	Shakdara (Tadjikistan)	Group 2
Ver-5	Verín (Spain)	Group 2

### 2.2 Quantification of virus multiplication

Virus multiplication was quantified as viral RNA accumulation 15 days post-inoculation via qRT-PCR and was used as a measure of plant resistance to virus infection. For each plant, four leaf disks of 4 mm in diameter from four systemically infected rosette leaves were collected. Total RNA extracts were obtained using TRIzol^®^ reagent (Life Technologies, Carlsbad, USA), and 0.32 ng of total RNA were added to the Brilliant III Ultra-Fast SYBR Green qRT-PCR Master Mix (Agilent Technologies, Santa Clara, USA) according to manufacturer’s recommendations. Specific primers were used to amplify a 70-nt fragment of the TuMV, and a 106-nt fragment of the CMV, coat protein (CP) gene, respectively (Lunello *et al.* 2007; Hily *et al.* 2014). Each sample was assayed by triplicate on a Light Cycler 480 II real-time PCR system (Roche, Indianapolis, USA). Absolute viral RNA accumulation was quantified as ng of viral RNA/μg of total RNA utilizing internal standards. For the two TuMV isolates, internal standards consisted in ten-fold dilution series of plasmid-derived RNA transcripts of the same 70 nt CP fragment from UK1-TuMV. For LS-CMV, ten-fold dilution series were prepared using purified viral RNA. Internal standards ranged from 2 × 10^−3^ng to 2 × 10^−7^ng.

### 2.3 Effect of infection on plant growth and reproduction

Aboveground plant structures were harvested at complete senescence. The weights of the rosette (*RW*), inflorescence (*IW*) and seeds (*SW*) were obtained. *RW* was used to estimate plant resources dedicated to growth, and *IW* and *SW* were utilized to estimate plant resources dedicated to reproduction (Thompson and Stewart 1981). The effect of virus infection on these traits was quantified by calculating infected to mock-inoculated plants ratios for each of them, dividing the value of each infected plant by the mean value of the mock-inoculated plants of the same genotype (*Trait_i_*/*Trait_m_*, *i* and *m* denoting infected and mock-inoculated plants, respectively). Following Pagán, Alonso-Blanco, and García-Arenal (2008), resource reallocation from growth to reproduction upon virus infection was analysed by calculating (*IW*/*RW*)_*i*_/(*IW*/*RW*)_*m*_ and (*SW*/*RW*)_*i*_/(*SW*/*RW*)_*m*_ ratios. Values of these ratios greater than one were considered as indicative of such resource reallocation. Seed viability, estimated as per cent germination, did not significantly differ between mock-inoculated (93.0–99.3%) and infected (91.0–99.7%) plants (*χ^2^* ≤ 2.16; *P *≥* *0.096). Also, virus infection did not affect the weight of a single seed (Wald *χ^2^* ≤ 0.99; *P *≥* *0.110) (Supplementary Table S3). Thus, *SW* similarly reflects the number of viable seeds in both mock-inoculated and infected plants.

### 2.4 Effect of infection on plant development

We recorded growth period (*GP*), as days from inoculation to the opening of the first flower; reproductive period (*RP*), as days from the opening of the first flower to the shattering of the first silique; and plant post-reproductive period (*PRP*), as days from the shattering of the first silique to plant senescence. In Arabidopsis, the opening of the first flower co-occurs with the end of the rosette growth, and the shattering of the first silique co-occurs with the end of flower production (Boyes *et al.* 2001). The total life period (*LP*) was quantified as the sum of the three periods. The effect of virus infection on *GP*, *RP* and *PRP*, was quantified as infected to mock-inoculated plants ratios. The (*RP*/*GP*)_*i*_/(*RP*/*GP*)_*m*_ and (*PRP*/*GP*)_*i*_/(*PRP*/*GP*)_*m*_ ratios were used to analyse virus-induced alterations of plant development.

### 2.5 Tolerance measure

Following Little *et al.* (2010) and Råberg (2014), range fecundity and mortality tolerances of each Arabidopsis genotype were calculated as the slope of the linear regression of *SW* and *LP*, respectively, to virus accumulation considering both infected and mock-inoculated plants.

### 2.6 Statistical analysis

First, we analysed the presence of outliers in the distribution of values of each trait by calculating the studentized residual for each data point, dividing the residual by its standard deviation. Values outside the 95 per cent CI of the Student t test distribution drawn with all of the studentized residuals were considered outliers (Sokal and Rohlf 1995). Analysed traits were not normally distributed, and variances were heterogeneous according to Kolmogorov–Smirnov and Levene’s tests, respectively. Virus accumulation was fitted to a lognormal distribution, *GP_i_*/*GP_m_*, *RP_i_*/*RP_m_*, *PRP_i_*/*PRP_m_* and *RW_i_*/*RW_m_* were fitted to a Weibull distribution; *LP_i_*/*LP_m_*, *IW_i_*/*IW_m_*, *SW_i_*/*SW_m_*, (*IW*/*RW*)_*i*_/(*IW*/*RW*)_*m*_ and (*SW*/*RW*)_*i*_/(*SW*/*RW*)_*m*_, and (*RP*/*GP*)_*i*_/(*RP*/*GP*)_*m*_ and (*PRP*/*GP*)_*i*_/(*PRP*/*GP*)_*m*_ were fitted to a Gamma distribution, and fecundity and mortality tolerance to a logistic distribution according to Akaike’s Information Criteria (R package: rriskDistributions; Belgorodski *et al.* 2015). Therefore, differences between viruses, plant genotypes and allometric groups/subgroups were analysed by generalized linear mixed models (GzLMMs) considering virus as fixed factor, and Arabidopsis genotype as random factor, which was nested to allometric group/subgroup (considered as fixed factor). Significant departure from zero of each trait value was analysed by simulating a dataset with zero mean and fitted to the same distribution than the corresponding trait and comparing the real and the simulated distribution using a Wilcoxon test. Trade-offs between resistance, fecundity tolerance and mortality tolerance were analysed using Spearman’s test, after outlier detection as described above. Tolerance–tolerance trade-offs according to virus and plant allometric group/subgroup were analysed using generalized linear models (GzLMs), considering both as fixed factors. Broad-sense heritability was estimated as $h_{b}^{2}$*= V_G_*/(*V_G_ + V_E_*), where *V_G_* is the among-genotypes variance component and *V_E_* is the residual variance. Variance components were determined using GzLMMs by the REML method (Lynch and Walsh 1998). GzLMMs and GzLMs were performed using R-libraries lme4, nlme and lmerTest (Bates *et al.* 2015, Kuznetsova, Brockhoff, and Christensen 2017, Pinheiro *et al.* 2018). Statistical analyses were conducted using R version 3.5.0 (R Core Team 2018).

## 3. Results

### 3.1 Virus multiplication in Arabidopsis

The level of UK1-TuMV, LS-CMV and JPN1-TuMV RNA accumulation was used to evaluate Arabidopsis resistance to virus infection. Accumulation differed according to the virus (Wald *χ^2^*_2,448_ = 211.52, *P *=* *1 × 10^−4^), and the interaction between virus and host genotype was significant (Wald *χ^2^*_34,448_ = 475.28, *P *<* *1 × 10^−4^). Thus, we analysed accumulation for each virus separately, considering plant genotype and allometric group as factors. For all three viruses, accumulation significantly differed between Arabidopsis genotypes (Wald *χ^2^* ≥ 137.17, *P *<* *1 × 10^−4^), but not between allometric groups (Wald *χ^2^* ≤ 0.41, *P *≥* *0.524) (Supplementary Table S1). Thus, the allometric group did not affect the level of resistance.

Broad-sense heritability of virus accumulation ranged from moderate to high depending on the virus: $h_{b}^{2}$ = 0.43, 0.60 and 0.68, for UK1-TuMV, LS-CMV and JPN1-TuMV, respectively (Supplementary Table S2). Therefore, there is significant genetic variation among the studied Arabidopsis genotypes for the ability to sustain virus multiplication.

### 3.2 Arabidopsis fecundity and mortality tolerance to virus infection

**Figure 2.**
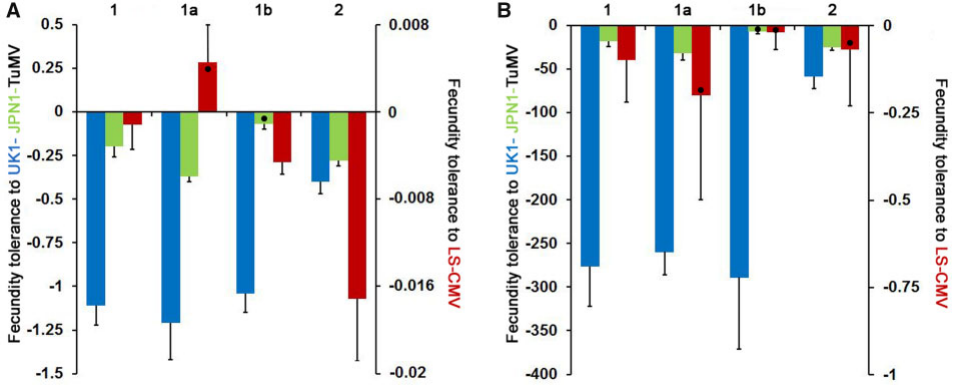
Arabidopsis fecundity and mortality tolerance to UK1-TuMV, LS-CMV and JPN1-TuMV. Panel A: values of fecundity tolerance to UK1-TuMV (blue), to JPN1-TuMV (green) and to LS-CMV (red) measured as the slope of the *SW* to virus accumulation linear regression. Panel B: values of mortality tolerance to the same three viruses measured as the slope of the *LP* to virus accumulation linear regression. Steeper slopes (lower values) indicate lower tolerance. Data are presented for allometric Groups 1 and 2, and for Subgroups 1a and 1b, and are mean ± standard errors across plant genotypes. Black dots indicate values non-different from zero. Note the different scales for tolerance to TuMV and to CMV.

Fecundity and mortality tolerances (slopes of the *SW* and *LP* to virus accumulation regression, respectively) differed depending on the virus (Wald *χ^2^*_2,48_ ≥ 143.28, *P *≤* *1 × 10^−4^). Overall, both tolerance measures were smallest to UK1-TuMV and greatest to LS-CMV, with tolerances to JPN1-TuMV showing intermediate values (Fig. 2 and Supplementary Table S1). The interaction between virus and Arabidopsis allometric group was also significant (Wald *χ^2^*_2,48_ ≥ 24.36, *P *≤* *1 × 10^−4^). Thus, fecundity and mortality tolerances were analysed for each virus independently. From here on, results will be presented firstly for the two viruses at the tolerance extremes (UK1-TuMV and LS-CMV), and lastly for the intermediate state (JPN1-TuMV).

**Figure 3.**
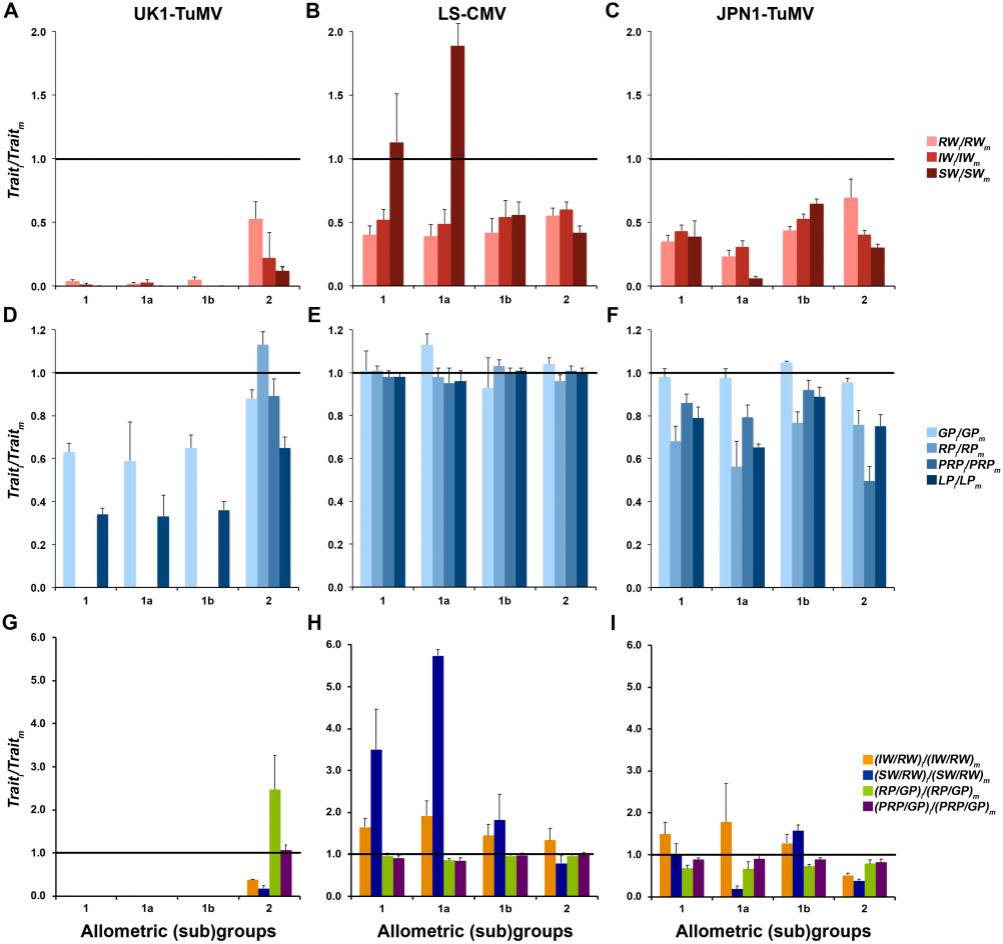
Effect of UK1-TuMV, LS-CMV and JPN1-TuMV infection on life-history traits for Arabidopsis allometric groups and subgroups. (A–C) Effect of viral infection on *RW*, *IW* and *SW*. (D–F) Effect of viral infection on *GP*, *RP* and *PRP*. (G–I) Effect of infection on the ratios *IW*/*RW*, *SW*/*RW*, *RP*/*GP* and *PRP*/*GP*. All effects were estimated as the ratio between infected (i) and mock-inoculated (m) plants. Data are presented for allometric Groups 1 and 2, and for Subgroups 1a and 1b, and are mean ± standard errors of plant genotype means. All values were different from zero except *IW* ratios for Group 1, and Subgroups 1a and 1b, in panel A.

In general, viruses significantly reduced Arabidopsis fecundity (*SW_i_*/*SW_m_* < 1) (Fig. 3). Exceptions were LS-CMV-infected Cum-0 and Ll-0 plants (Group 1) that overcompensated the effect of virus infection (*SW_i_*/*SW_m_* > 1) (Supplementary Table S1). Upon UK1-TuMV infection, fecundity tolerance varied according to the allometric group (Wald *χ*^2^_1,16_ = 23.68, *P *<* *1 × 10^−4^). The negative slope was stepper for Group 1 genotypes, with none of the infected plants producing seeds, than for Group 2 ones, with only 61.8 per cent of sterilized plants and fertile individuals in 8/11 genotypes (Fig. 2 and Supplementary Table S1). Thus, Group 2 plants had higher fecundity tolerance. All LS-CMV-infected plants were fertile, with stepper negative slopes of the *SW* to virus accumulation regression for Group 2 genotypes (lower fecundity tolerance) than for Group 1 ones (Wald *χ*^2^_1,16_ = 12.34, *P *<* *1 × 10^−4^). At odds with UK1-TuMV and LS-CMV-infected plants, in JPN1-TuMV-infected plants the slope of the *SW* to virus accumulation regression did not differ between allometric groups (Wald *χ*^2^_1,16_ = 0.83, *P *=* *0.362). However, Group 1 genotypes showed a bimodal response, and were divided into two subgroups according to this response: In Cum-0, Kas-0 and Ll-0 (Subgroup 1a), 50–70 per cent of infected individuals were sterilized and regression slopes were steep. Conversely, infected Cad-0, Cdm-0, Kas-2 and Kyo-1 plants (Subgroup 1b) produced seeds and regression slopes were shallower than for Subgroup 1a (Wald *χ*^2^_1,5_ = 46.19, *P *<* *1 × 10^−4^) (Fig. 2 and Supplementary Table S1). Group 2 genotypes, where 92 per cent of infected plants produced seeds, showed intermediate and significantly different slope values than the two Group 1 subsets (Wald *χ^2^* ≥ 4.61, *P *≤* *0.040) (Fig. 2).

UK1-TuMV also reduced plant survival (*LP_i_*/*LP_m_* < 1) (Fig. 3), with mortality tolerance differing between allometric groups (Wald *χ*^2^_1,16_ = 29.69, *P *=* *1 × 10^−4^). Mortality tolerance was smaller (steeper negative slope of the *LP* to virus accumulation regression) for Group 1 than for Group 2 plants (Wald *χ*^2^_1,16_ = 29.69, *P *=* *1 × 10^−4^) (Fig. 2). In contrast, LS-CMV infection had little effect on Arabidopsis survival: No differences between allometric groups were observed in the slope of the *LP* to virus accumulation regression (Wald *χ*^2^_1,16_ = 0.02, *P *=* *0.900), indicating similar mortality tolerance, with very little effect of virus infection on *LP* as denoted by *LP_i_*/*LP_m_* values near one (Figs 2 and 3), and slopes not different from zero (*W* ≤ −0.439; *P *≥* *0.570). As for JPN1-TuMV-infected plants, mortality tolerance did not vary between allometric groups (Wald *χ*^2^_1,16_ = 1.25, *P *=* *0.263). However, again Group 1 plants presented a bimodal response to infection: the slope of the *LP* to virus accumulation regression was significantly steeper in Subgroup 1a than in Subgroup 1b (Wald *χ*^2^_1,5_ = 14.25, *P *=* *1 × 10^−3^) and in Group 2 genotypes (Wald *χ*^2^_1,13_ = 8.34, *P *=* *0.004), which showed similar mortality tolerance (Wald *χ*^2^_1,13_ = 0.83, *P *=* *0.363) (Fig. 2 and Supplementary Table S1). Note that upon infection by UK1-TuMV and LS-CMV Group 1 genotypes did not show this bimodal distribution (Wald *χ^2^* ≤ 0.49, *P *≥* *0.483) (Supplementary Table S1).

Because fecundity and mortality tolerances are genotype-specific rather than plant-specific variables, by definition heritability for these traits could not be calculated.

#### 3.3 Relationship between modifications of Arabidopsis life-history traits and tolerance to virus infection

For each virus, the effect of infection on Arabidopsis growth and reproduction was quantified as the ratios of rosette, inflorescence and seed weights between infected and mock-inoculated plants (*RW_i_*/*RW_m_*, *IW_i_*/*IW_m_* and *SW_i_*/*SW_m_*, respectively) (Fig. 3A–C and Supplementary Table S1). In general, virus infection reduced *RW*, *IW* and *SW* (Wald *χ^2^* ≥ 49.52; *P *<* *1 × 10^−4^), this reduction always depending on the Arabidopsis genotype (Wald *χ^2^* ≥ 388.79; *P *<* *1 × 10^−4^). For UK1-TuMV-infected plants, all ratios also depended on the allometric group (Wald *χ*^2^_1,168_ ≥ 25.87; *P *<* *1 × 10^−4^). In Groups 1 and 2, *RW_i_*/*RW_m_* was greater than *IW_i_*/*IW_m_* (Wald *χ^2^* ≥ 20.04; *P *<* *1 × 10^−4^) and *SW_i_*/*SW_m_* (Wald *χ^2^* ≥ 50.20; *P *<* *1 × 10^−4^) suggesting no resource reallocation from growth to reproduction. Indeed, (*IW*/*RW*)_*i*_/(*IW*/*RW*)_*m*_ and (*SW*/*RW*)_*i*_/(*SW*/*RW*)_*m*_ were always smaller than one (Wald *χ*^2^_1,168_ ≥ 21.35, *P *<* *1 × 10^−4^) (Fig. 3G and Supplementary Table S1). The effect of LS-CMV on *RW* and *SW* (Wald *χ*^2^_1,163_ ≥ 4.33, *P *≤* *0.037), but not on *IW* (Wald *χ*^2^_1,163_ = 1.955, *P *=* *0.162), varied according to the allometric group. For Group 1, the effect of LS-CMV infection on *RW* was larger than on *SW* (Wald *χ*^2^_1,49_ = 10.89, *P *=* *1 × 10^−3^), whereas the opposite was observed in Group 2 (Wald *χ*^2^_1,102_ = 13.90, *P *=* *2 × 10^−4^). (*SW*/*RW*)_*i*_/(*SW*/*RW*)_*m*_ differed between allometric groups (Wald *χ*^2^_1,163_ = 11.77, *P *<* *1 × 10^−4^), values being greater than one only for Group 1 (Wald *χ*^2^_1,60_ = 7.11, *P *=* *0.008) (Fig. 3H and Supplementary Table S1). Similar trends were observed in JPN1-TuMV-infected plants (Fig. 3C), for which (*IW*/*RW*)_*i*_/(*IW*/*RW*)*_m_* (Wald *χ*^2^_1,161_ = 2.66, *P *=* *0.003) and (*SW*/*RW*)_*i*_/(*SW*/*RW*)*_m_* (Wald *χ*^2^_1,161_ = 17.18, *P *<* *1 × 10^−4^) were also greater for Group 1 than for Group 2 plants, values being similar or greater than one only for Group 1 (Fig. 3I and Supplementary Table S1). These results would be compatible with resource reallocation from growth to reproduction in LS-CMV- and JPN1-TuMV-infected Group 1 plants. Again, JPN1-TuMV-infected Group 1 genotypes showed a bimodal distribution: *RW_i_*/*RW_m_*, *IW_i_*/*IW_m_* and *SW_i_*/*SW_m_* were smaller for Subgroup 1a than for Subgroup 1b (Wald *χ*^2^_1,161_ ≥ 5.65, *P *≤* *0.017) (Fig. 3C), and the same was observed for (*SW*/*RW*)_*i*_/(*SW*/*RW*)*_m_* (Wald *χ*^2^_1,161_ = 5.76, *P *=* *0.016) (Fig. 3I). This ratio was greater than one only for Subgroup 1b genotypes (Wald *χ*^2^_1,35_ = 80.95, *P *<* *1 × 10^−4^), indicating that resource reallocation was associated with fecundity tolerance in this subgroup (Fig. 3I and Supplementary Table S1).

We also quantified the effect of infection on the plant growth, reproductive and post-reproductive periods (*GP_i_*/*GP_m_*, *RP_i_*/*RP_m_* and *PRP_i_*/*PRP_m_*, respectively) (Fig. 3D–F and Supplementary Table S1). Upon UK1-TuMV infection, *GP_i_*/*GP_m_* depended on the allometric group (Wald *χ*^2^_1,166_ = 17.95, *P *<* *1 × 10^−4^), this ratio being smaller for Group 1 than for Group 2 plants. Interestingly, in Group 2 genotypes the effect of infection on *GP* was greater than the effect on *RP* (Wald *χ*^2^_1,39_ = 52.46, *P *<* *1 × 10^−4^): *GP_i_*/*GP_m_* was significantly smaller (Wald *χ*^2^_1,39_ = 9.73, *P *=* *0.002), and *RP_i_*/*RP_m_* greater (Wald *χ*^2^_1,39_ = 7.55, *P *=* *0.006), than one. Thus, upon UK1-TuMV infection more tolerant Group 2 genotypes shortened their growth period but elongated the time dedicated to reproduction, as indicated by (*RP*/*GP*)_*i*_/(*RP*/*GP*)_*m*_ values greater than one in this subgroup (Fig. 3G and Supplementary Table S1). Note that in Group 1 genotypes *RP* and *PRP* could not be quantified because plants did not produce mature siliques (Fig. 3D and Section 2). On the other hand, LS-CMV infection did not affect *GP*, *RP* and *PRP* (Wald *χ*^2^_1,166_ ≤ 1.94, *P *≥* *0.164) their ratios being always near one in both allometric groups (Wald *χ^2^* ≤ 0.76, *P *≥* *0.383) (Fig. 3E). Accordingly, (*RP/GP*)*_i_/*(*RP/GP*)_*m*_ and (*PRP/GP*)*_i_/*(*PRP/GP*)_*m*_ were also near one (Wald *χ^2^* ≤ 2.47, *P *≥* *0.116) (Fig. 3H and Supplementary Table S1). Exception to this rule was Subgroup 1a, which included Arabidopsis genotypes that overcompensated the effect of LS-CMV infection on *SW*. These genotypes significantly elongated *GP*, as indicated by *GP_i_/GP_m_* values higher (Wald *χ*^2^_1,59_ = 11.885, *P *=* *6 × 10^−4^), and (*RP/GP*)_i_/(*RP/GP*)_*m*_ and (*PRP/GP*)_i_/(*PRP/GP*)_m_ values smaller (Wald *χ*^2^_1,59_ ≥ 6.77, *P *≤* *0.009), than one. Finally, in JPN1-TuMV-infected plants *GP_i_/GP_m_*, *RP_i_/RP_m_* and *PRP_i_/PRP_m_* did not depend on the allometric group (Wald *χ*^2^_1,166_ ≤ 0.88, *P *≥* *0.349) (Fig. 3F and Supplementary Table S1). Also, (*RP/GP*)_i_/(*RP/GP*)_*m*_ and (*PRP/GP*)_i_/(*PRP/GP*)_m_ did not differ between Groups 1 and 2 and showed values smaller than one (Wald *χ*^2^ ≤ 0.52, *P *≥* *0.470) (Fig. 3I and Supplementary Table S1). The effect of infection on all plant developmental traits was similar in Subgroups 1a and 1b (Wald *χ*^2^_1,166_ ≤ 3.77, *P *≥* *0.070).

In summary, Arabidopsis fecundity and mortality tolerances to UK1-TuMV are associated with modifications of the plant developmental schedule, whereas fecundity tolerance to LS-CMV and JPN1-TuMV is accompanied by resource reallocation from growth to reproduction. Interestingly, broad-sense heritability of the effect of UK1-TuMV infection on *GP*, *RP* and *PRP* was higher than that of the effect of infection on *RW* and *IW* ($h_{b}^{2}$ = 0.58–0.83 vs. 0.40–0.58), whereas the opposite was observed for LS-CMV and JPN1-TuMV-infected plants ($h_{b}^{2}$=0.17–0.39 vs. 0.38–0.41 and 0.50–0.61 vs. 0.68–0.87, respectively) (Supplementary Table S2). Thus, the plant life-history traits associated with the tolerance response to a given virus have higher host dependency that those not related to tolerance to that particular virus.

### 3.4 Trade-offs between Arabidopsis defences to virus infection

To analyse Arabidopsis resistance-tolerance trade-offs to each virus, bivariate relationships between virus accumulation and the slope of the *SW* and *LP* to virus accumulation regression were explored, a significantly negative association indicating a trade-off. No significantly negative association was observed between resistance and the two measures of tolerance for any of the three viruses, neither using the whole set of plant genotypes (*r *≤* *0.23; *P *≥* *0.367), nor for each allometric group (*r *≤* *0.40; *P *≥* *0.223).

**Figure 4.**
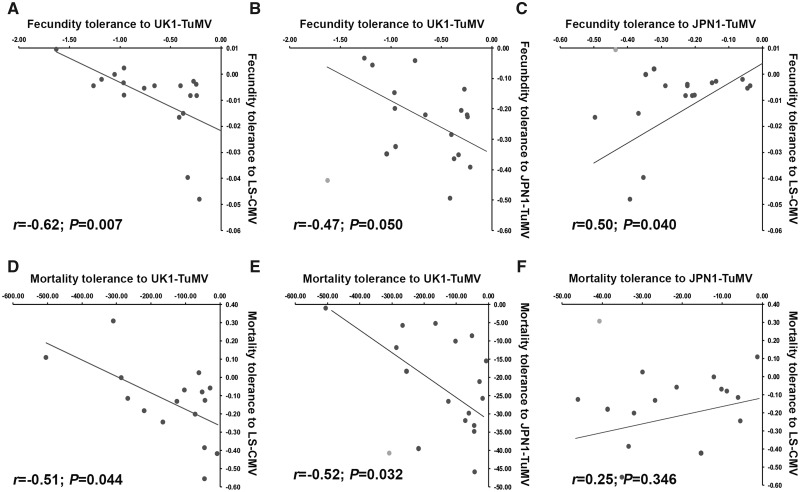
Trade-offs between Arabidopsis tolerances to UK1-TuMV, LS-CMV and JPN1-TuMV. (A–C) Pairwise linear regressions between fecundity tolerance to UK1-TuMV, LS-CMV and JPN1-TuMV. (D–F) Pairwise linear regressions between mortality tolerance to UK1-TuMV, LS-CMV and JPN1-TuMV. Data are slope of the *SW* (fecundity tolerance) and *LP* (mortality tolerance) to virus accumulation regression for each Arabidopsis genotype. Grey dots correspond to outlier values, which were excluded from the analyses.

We used the same approach to analyse fecundity tolerance-tolerance trade-offs (Fig. 4A–C). Bivariate analyses indicated a negative relationship between fecundity tolerance to UK1-TuMV and to LS-CMV (*r* = −0.62; *P *=* *0.007). No association was found between fecundity tolerance to UK1-TuMV and to JPN1-TuMV (*r* = −0.20; *P *=* *0.418), but this was just due to a single outlier value (*r* = −0.47; *P *=* *0.050). Finally, no association was detected between fecundity tolerance to JPN1-TuMV and LS-CMV (*r *=* *0.25; *P *=* *0.325), but again removal of a single outlier resulted in a positive association (*r *=* *0.50; *P *=* *0.040) (Fig. 4A–C). In both cases, outlier values corresponded to Ll-0. Because our previous results strongly suggested that trade-offs between tolerance to different viruses were linked to plant allometry, we also analysed such trade-offs by GzLMs virus pairwise comparisons of the slope of the *SW* to virus accumulation regression considering virus and allometric group as factors. A significant interaction was taken as indicative of a tolerance–tolerance trade-off. When fecundity tolerance upon UK1-TuMV infection was compared with that upon infection by the other two viruses, a significant virus per allometric group interaction was observed (Wald *χ*^2^*≥* 35.12, *P *<* *1 × 10^−4^). On the other hand, the virus genotype per allometric group interaction was not significant when comparing JPN1-TuMV and LS-CMV (Wald *χ*^2^_1,34_*=* 1.87, *P *=* *0.275) (Fig. 2A). Given the bimodal distribution of fecundity tolerance in Group 1 JPN1-TuMV-infected plants, we also performed pairwise comparisons considering Subgroups 1a and 1b. When fecundity tolerance upon UK1-TuMV and LS-CMV infection was compared between Subgroups 1a and 1b, and Group 2, a significant interaction between factors was observed (Wald *χ*^2^_1,28_*≥* 24.89, *P *<* *1 × 10^−4^) (Fig. 2A). The comparison of JPN1-TuMV- and LS-CMV-infected plants yielded a significant interaction only between Subgroup 1a and Group 2 (Wald *χ*^2^_1,28_*=* 12.34, *P *=* *4 × 10^−4^) (Fig. 2A). Conversely, the comparison of UK1-TuMV- and JPN1-TuMV-infected plants indicated a significant interaction between virus genotype and plant allometry for the combination of Subgroup 1b and Group 2 (Wald *χ*^2^_1,28_*=* 35.97, *P *<* *1 × 10^−4^) (Fig. 2A). Altogether, these results indicate trade-offs between fecundity tolerance to UK1-TuMV and to the other two viruses.

Bivariate analyses indicated a significant negative association between mortality tolerance to UK1-TuMV and to LS-CMV (*r* = −0.51; *P *=* *0.044), and between tolerance to UK1-TuMV and to JPN1-TuMV when excluding a single outlier (*r* = −0.52; *P *=* *0.032). No significant association was observed between mortality tolerance to JPN1-TuMV and to LS-CMV (*r *=* *0.12; *P *=* *0.627 and *r *=* *0.25; *P *=* *0.346, with and without considering outliers, respectively) (Fig. 4D–F). Again, in both cases outliers corresponded to Ll-0 values. In addition, the comparison of slope of the *LP* to virus accumulation regression between plants infected by UK1-TuMV and by the other two viruses showed a significant virus per allometric group interaction (Wald *χ*^2^*≥* 29.69, *P *<* *1 × 10^−4^), whereas no such interaction was detected between JPN1-TuMV and LS-CMV (Wald *χ*^2^_1,34_*=* 1.26, *P *=* *0.261) (Fig. 2B). When Group 1 plants were divided into Subgroups 1a and 1b, the only significant interaction was between Subgroup 1b and Group 2 genotypes for comparisons of mortality tolerance to UK1-TuMV and JPN1-TuMV (Wald *χ*^2^_1,29_*=* 22.35, *P *<* *1 × 10^−4^) (Fig. 2B). These results indicate trade-offs between mortality tolerance to UK1-TuMV and to the other two viruses.

For each virus, we also analysed potential mortality-fecundity tolerance trade-offs. No significant bivariate associations were found when considering all plant genotypes together, or each allometric group separately in any of the three viruses (*r *≤* *0.64; *P *≥* *0.119). Exception were UK1-TuMV-infected plants when analysed as a whole, in which both tolerances showed a positive association (*r *=* *0.57; *P *=* *0.013). Thus, when associated, higher mortality tolerance increases fecundity tolerance to a given virus.

## 4. Discussion

Accumulating evidence indicates that tolerance is as widespread as resistance as a plant defence strategy, and therefore central to understand plant–pathogen (including plant–virus) interactions. However, the mechanisms by which tolerance is achieved and the forces shaping its evolution are still poorly understood (Baucom and de Roode 2011; Pagán and García-Arenal 2018). Using Arabidopsis and its natural pathogens CMV and TuMV, we tested the hypotheses that tolerances to pathogens with different virulence levels are associated with modifications of different plant life-history traits, and that the evolution of tolerance to a given pathogen depends on trade-offs established with the level of tolerance to others. For this purpose, we used two TuMV and one CMV isolate, none of them obtained from Arabidopsis. It could be argued that this may limit the conclusions of our work on the ecological and evolutionary implications of the observed tolerance responses. However, it should be noted that our previous work indicates that adaptation of JPN1-TuMV to Arabidopsis resulted in an infection phenotype similar to that of UK1-TuMV (Vijayan *et al.* 2017), which suggests that isolates adapted to Arabidopsis would induce host responses similar to those of UK1-TuMV. JPN1-TuMV would represent the case of virus cross-species transmission from other Brassicaceae hosts, which are known to co-exist with natural Arabidopsis populations (McLeish *et al.* 2019). On the other hand, LS-CMV has been shown to induce comparable tolerance responses than other CMV isolates, including some obtained from Arabidopsis, with virus isolate explaining a minimal proportion of the Arabidopsis variance in tolerance (Pagán, Alonso-Blanco, and García-Arenal 2007; Montes, Alonso-Blanco, and García-Arenal 2019). This is not surprising given the generalist nature of CMV (it infects more than 1,200 plant species, including host species that share habitat with Arabidopsis), which makes unlikely that CMV isolates from Arabidopsis co-evolved for a long time with the host. Therefore, the selected isolates reasonably reflect what could be expected in nature.

We showed that Arabidopsis displays genotype-specific fecundity tolerance to the highly virulent virus UK1-TuMV, with plants of the allometric Group 2 having higher tolerance than Group 1 ones. In Arabidopsis, UK1-TuMV infection often prevents seed production (Sánchez *et al.* 2015; Vijayan *et al.* 2017; this work), such that this virus can be considered as a sterilizing pathogen. Because sterilizing pathogens have an enormous impact on the host fitness, hosts are expected to evolve defences against this type of pathogens (Lafferty and Kuris 2009). Theoretical models on the evolution of host defences predict that infection by a sterilizing pathogen promotes tolerance rather than resistance. Resistance restricts pathogen multiplication and the evolution of resistance has a high cost in terms of host fitness as preventing parasite multiplication drains all resources that should be devoted to reproduction. As a consequence, if the host wants to evolve resistance must pay the enormous cost of reducing its fitness to zero. Conversely, tolerance would compensate the effect of infection without attempting to control pathogen’s multiplication, thus being less costly (Restif and Koella 2004; Best, White, and Boots 2010). Although we did not analyse the costs of resistance and tolerance, our results would support this prediction in that Arabidopsis evolves tolerance to a sterilizing virus rather than resistance: First, half of the Arabidopsis genotypes were not sterilized by UK1-TuMV regardless of virus multiplication, which by definition increases tolerance. Second, the level of resistance did not relate with plant fitness (i.e. infected plants of the two allometric groups sustained similar multiplications levels but greatly differed in seed production), and extreme resistance (immunity) was not detected, indicating that resistance is not associated with the effect of UK1-TuMV on progeny production.

It should be noted that upon UK1-TuMV infection, infected plants of tolerant Arabidopsis genotypes produced on average 30 per cent of the seeds produced by mock individuals. It could be argued that this level of fecundity tolerance is not effective, i.e. seed production of infected plants is far from that of uninfected ones (Shuckla, Pagán, and García-Arenal 2018). However, mathematical models on the evolution of tolerance to sterilizing pathogens predict that optimal levels of tolerance will not surpass 50 per cent of the progeny produced by uninfected individuals, regardless of tolerance being modelled as a function of host mortality, lifespan or transmission rate (Restif and Koella 2004; Hall, Becker, and Caceres 2007; Best, White, and Boots 2010). Even if we consider 30 per cent of progeny production upon UK1-TuMV infection as a low level of fecundity tolerance, it would be selectively advantageous for Arabidopsis, as it makes the difference between leaving progeny or not. Indeed, various models showed that this level of fecundity tolerance drives the host population out of the pathogen-driven extinction margins, especially at high levels of pathogen prevalence (Boots and Sasaki 2002; Antonovics 2009). Accordingly, experimental analyses in other host-sterilizing pathogen interactions reported similar fecundity tolerance levels (Fredensborg and Poulin 2006; Vale and Little 2012). It is relevant to mention that Arabidopsis fecundity and mortality tolerances to UK1-TuMV were positively associated, whereas upon infection by milder viruses they were not. This observation would agree with models predicting that, for highly virulent parasites, fecundity tolerance is a saturating function of mortality tolerance (Best, White, and Boots 2010), provided that our data is in the linear part of the curve. Moreover, this virus-specific association between fecundity and mortality tolerance indicates that the observed relationship is not due to some accessions performing generally better than others in our particular greenhouse conditions. In such case, we would have expected to find a general positive association between both tolerance measures. Altogether, to our knowledge these results would represent the first example of plant tolerance to a sterilizing virus.

Fecundity tolerance to UK1-TuMV was associated with genotype-specific modifications of the plant developmental schedule. Particularly, upon UK1-TuMV infection more fecundity-tolerant Group 2 genotypes showed shorter growth, and longer reproductive, periods than mock-inoculated plants. This observation agrees with the prediction of the life-history theory that bringing forward the age at maturity allows infected hosts to reproduce before they experience the full cost of infection, thus compensating (at least partly) the effect on host fitness (Hochberg, Michalakis, and de Meeus 1992; Gandon, Agnew, and Michalakis 2002). These results are also in agreement with experimental analyses of life-history modifications upon infection by highly virulent parasites in animals (e.g. Agnew, Koella, and Michalakis 2000; Ebert *et al.* 2004; Fredensborg and Poulin 2006). Bringing forward the age at maturity may have important consequences for Arabidopsis population dynamics. Early progeny production would allow seeds from infected plants to germinate and occupy the most suitable niches before uninfected individuals produce theirs, which represents a competitive advantage (Akiyama and Ågren 2014; Gioria, Pyšek, and Osborne 2018). This could contribute to compensate the smaller progeny production of infected plants, provided that virus infection does not affect seed viability as shown here. Shorter growth, and longer reproductive, periods of Group 2 genotypes were also associated with higher mortality tolerance to UK1-TuMV. It has been shown that, in Arabidopsis, inflorescences contribute to lifetime carbon gain more than rosettes, particularly for accessions with smaller rosettes and larger inflorescences as those in Group 2 (Earley *et al.* 2009). Thus, plants that produce inflorescence earlier, and maintain them for longer, would have greater carbon budget, which would contribute to reduce the effect of virus infection on plant survival without limiting pathogen multiplication (i.e. mortality tolerance). Indeed, increased carbon fixation has been associated with plant mortality tolerance in other plant–pathogen interactions (reviewed by Pagán and García-Arenal 2018). Interestingly, this mechanism would also explain how Group 2 plants achieve fecundity tolerance to UK1-TuMV even in the absence of resource reallocation.

Arabidopsis fecundity tolerance to LS-CMV was higher in Group 1 than in Group 2 genotypes, which was associated with resource reallocation from growth to reproduction, an extensively studied response (Pagán, Alonso-Blanco, and García-Arenal 2007, 2008, 2009, Hily *et al.* 2014, 2016; Shuckla, Pagán, and García-Arenal 2018). Notably, our results are in agreement with these previous works even if we quantified tolerance as the slope of the fitness to virus load regression rather than at a single pathogen load, and support the life-history theory prediction that hosts would evolve tolerance to milder pathogens (as CMV) through resource reallocation from growth to reproduction (Hochberg, Michalakis, and de Meeus 1992; Gandon, Agnew, and Michalakis 2002). Thus, it could be concluded that Arabidopsis tolerance to plant virus infection is virulence dependent, which is another prediction of the life-history theory. However, our results could be also explained if Arabidopsis life-history trait modifications were virus species specific, rather than depend on virulence. Indeed, using six Arabidopsis genotypes and LS-CMV, Shuckla, Pagán, and García-Arenal (2018) concluded that fecundity tolerance through resource reallocation was specific to CMV, but these authors only considered the highly virulent UK1-TuMV isolate. The effect of a milder TuMV genotype (JPN1-TuMV) on Arabidopsis might shed light on this question. Upon JPN1-TuMV infection, half of the Group 1 genotypes showed higher mortality and fecundity tolerances than Group 2 genotypes, all infected plants being fertile, and tolerance being associated with resource reallocation from growth to reproduction. In the other half of Group 1 genotypes, JPN1-TuMV sterilized over 50 per cent of the plants and no tolerance response was observed. Therefore, Arabidopsis Group 1 genotypes in which JPN1-TuMV infection has lower virulence display similar responses to those observed upon LS-CMV infection, whereas in plant genotypes for which JPN1-TuMV virulence is higher the effect of infection resembles to that of UK1-TuMV. This strongly suggests that tolerance is virulence dependent rather than virus specific. Note that the subdivision of Group 1 genotypes resulted in three to four genotypes per subgroup, and the generality of our observations should be validated in a larger number of Arabidopsis genotypes, and in other pathogens and hosts.

We failed in finding a negative association between plant resistance and tolerance to the same virus across Arabidopsis genotypes, which indicates the absence of trade-offs between these two defence mechanisms in agreement with previous analyses (e.g. Simms and Triplett, 1994; Kover and Schaal, 2002; Carr, Murphy, and Eubanks 2006; Montes, Alonso-Blanco, and García-Arenal 2019). On the other hand, Arabidopsis could not optimize at the same time tolerances to viruses displaying different virulence levels (negative association between these tolerances), with LS-CMV and JPN1-TuMV (lower virulence) inducing different and mutually exclusive life-history modifications than UK1-TuMV (higher virulence). In contrast, fecundity tolerances to LS-CMV and JPN1-TuMV, with similar virulence levels, were simultaneously maximized (positive association between these tolerances) through similar life-history modifications. These results are indicative of trades-offs between tolerances to viruses with different virulence levels and suggest that tolerance mechanisms to milder viruses are linked. Interestingly, the negative association between tolerances was greater for the UK1-TuMV vs. LS-CMV than for the UK1-TuMV vs. JPN1-TuMV combination. This suggests that such trade-offs may vary in strength along a continuum (i.e. from very strong to non-existent) depending on the viruses involved. In addition, our analyses showed that, even when trade-offs are detected, certain accessions (e.g. Ll-0) depart from the general trend, suggesting that not every accession may display such trade-offs. Together, these observations are indicative that the tolerance trade-offs reported here might not be pervasive but depend on the host and virus genotypes involved. Analyses in other plant–virus–virus interactions will be necessary to tests this hypothesis.

A number of experimental works reported that pathogen-driven changes in host life-history traits can be either genetically determined or the consequence of phenotypic plasticity (Michalakis and Hochberg 1994; Schlichting and Pigliucci 1998; McLeod and Day 2015). Thus, it could be hypothesized that one or both of these two types of determinisms may be involved in the observed tolerance-tolerance trade-offs. Our data indicates that trade-offs are influenced by two main factors: 1, Virus virulence: plant genotypes showed different responses in different environments (i.e. virulence levels), which is indicative of phenotypic plasticity (Michalakis and Hochberg 1994). 2, Plant allometry: Group 1 genotypes showed tolerance to less virulent viruses through resource reallocation, whereas Group 2 genotypes showed tolerance to the most virulent one by altering plant development. Arabidopsis Group 1 genotypes have bigger rosettes and smaller inflorescences than Group 2 ones. That is, in Group 1 genotypes most resources are diverted into growth, whereas in Group 2 resources are primarily dedicated to reproduction. Hence, Group 1 plants would have a relatively wide margin to reallocate growth resources into reproduction; this margin being much narrower, and therefore less efficient, for Group 2 genotypes. In addition, bringing forward the age at maturity requires accelerated rosette growth rates, as Arabidopsis needs to reach a minimum rosette size to flower (Méndez-Vigo 2010). Group 1 genotypes typically show faster rosette growth rates (Hily *et al.* 2016), and therefore have less margin to accelerate it, than Group 2 genotypes. Thus, the two allometric groups have particular characteristics that are genetically determined (Manzano-Piedras *et al.* 2014), and that could influence the evolution of tolerance. In support of this genetic determinism, our results indicated that heritability in tolerance-related plant traits was always medium to high. Therefore, although fecundity tolerance is a phenotypically plastic response, the type of response depends on the genetic background of the plant, and tolerance–tolerance trade-offs likely have both genetic and phenotypic plasticity components. This combination of phenotypic plasticity and genetic determinism for tolerance has been also shown in response to other factors such as the moment of plant inoculation, light, temperature and plant density (Pagán, Alonso-Blanco, and García-Arenal 2007, 2009; Hily *et al.* 2016; Montes and Pagán 2019), factors that would modulate the tolerance–tolerance trade-offs observed here, which would be an interesting avenue for future research.

Tolerance–tolerance trade-offs may have important implications for understanding the evolution of host defences. To date, most mathematical models on this subject are built on the assumption that tolerance evolves in single host–pathogen interactions (Kutzer and Armitage 2016; Pagán and García-Arenal 2018). These models predict that tolerance would be selectively advantageous for both the host and the pathogen, as tolerance will increase its prevalence, such that genes conferring tolerance will become fixed in the host population (Rausher 2001; Råberg, Graham, and Read 2009). This is generally applicable to mortality tolerance because it increases the infectious period but would only apply to fecundity tolerance if the pathogen is vertically transmitted (Best, White, and Boots 2008). In Arabidopsis, CMV and TuMV are seed transmitted (Pagán *et al.* 2014; Montes and Pagán 2019). However, our data suggest polymorphisms for both fecundity and mortality tolerance. Increasing evidence indicate that in nature host populations are invaded by more than one pathogen, occurring in single and mixed infections (Syller 2012). Thus, host defences often evolve in a multi-pathogen context. Our results indicate that, in this scenario, the evolution of both fecundity and mortality tolerance to a given virus comes at the cost of higher susceptibility to other(s), which may impose a selection pressure on tolerance and prevent fixation. Hence, more realistic analyses on the evolution of host defences should consider the combined effects of more than one pathogen, and not necessarily in coinfection.

## Supplementary Material

veaa019_Supplementary_Data

## Data Availability

Data are available in Supplementary material.

## References

[veaa019-B1] Agnew P., Koella J. C., Michalakis Y. (2000) ‘Host Life History Responses to Parasitism’, Microbes and Infection, 2: 891–6. 10962272 10.1016/s1286-4579(00)00389-0

[veaa019-B2] Akiyama R., Ågren J. (2014) ‘Conflicting Selection on the Timing of Germination in a Natural Population of *Arabidopsis thaliana*’, Journal of Evolutionary Biology, 27: 193–9. 24329869 10.1111/jeb.12293

[veaa019-B3] Anderson P. K. et al (2004) ‘Emerging Infectious Diseases of Plants: Pathogen Pollution, Climate Change and Agrotechnology Drivers’, Trends in Ecology & Evolution, 19: 535–44. 16701319 10.1016/j.tree.2004.07.021

[veaa019-B4] Antonovics J. (2009) ‘The Effect of Sterilizing Diseases on Host Abundance and Distribution along Environmental Gradients’, Proceedings of the Royal Society B: Biological Sciences, 276: 1443–8. 10.1098/rspb.2008.1256 PMC2677222 19324815

[veaa019-B5] Bates D. et al (2015) ‘Fitting Linear Mixed-Effects Models Using lme4’, Journal of Statistic Software, 67: 1–48.

[veaa019-B6] Baucom R. S., de Roode J. C. (2011) ‘Ecological Immunology and Tolerance in Plants and Animals’, Functional Ecology, 25: 18–28.

[veaa019-B7] Belgorodski N. et al 2015 *rriskDistributions: Fitting Distributions to Given Data or Known Quantiles*, R Package Version 2.1.2 <https://CRAN.R-project.org/package=rriskDistributions> accessed 26 Aug 2018.

[veaa019-B8] Best A., White A., Boots M. (2008) ‘Maintenance of Host Variation in Tolerance to Pathogens and Parasites’, Proceedings of the National Academy of Sciences of the United States of America, 105: 20786–91. 19088200 10.1073/pnas.0809558105 PMC2634923

[veaa019-B9] Best A., White A., Boots M. (2010) ‘Resistance is Futile but Tolerance Explains Why Parasites Do Not Castrate Their Hosts’, Evolution, 64: 348–57. 19686267 10.1111/j.1558-5646.2009.00819.x

[veaa019-B10] Boots M., Bowers R. G. (1999) ‘Three Mechanisms of Host Resistance to Microparasites—Avoidance, Recovery and Tolerance—Show Different Evolutionary Dynamics’, Journal of Theoretical Biology, 201: 13–23. 10534432 10.1006/jtbi.1999.1009

[veaa019-B11] Boots M., Sasaki A. (2002) ‘Parasite-Driven Extinction in Spatially Explicit Host–Parasite Systems’, The American Naturalist, 159: 706–13. 10.1086/339996 18707391

[veaa019-B12] Boyes D. C. et al (2001) ‘Growth Stage–Based Phenotypic Analysis of Arabidopsis: A Model for High Throughput Functional Genomics in Plants’, The Plant Cell, 13: 1499–510. 11449047 10.1105/TPC.010011 PMC139543

[veaa019-B13] Carr D. E., Murphy J. F., Eubanks M. D. (2006) ‘Genetic Variation and Covariation for Resistance and Tolerance to *Cucumber mosaic virus* in *Mimulus guttatus* Phrymaceae: A Test for Costs and Constraints’, Heredity, 96: 29–38. 16189544 10.1038/sj.hdy.6800743

[veaa019-B14] Clarke D. D. (1986) ‘Tolerance of Parasites and Disease in Plants and Its Significance in Host-Parasite Interactions’, Advances in Plant Pathology, 5: 161–98.

[veaa019-B15] Day T. (2002) ‘On the Evolution of Virulence and the Relationship between Various Measures of Mortality’, Proceedings of the Royal Society of London. Series B: Biological Sciences, 269: 1317–23. 12079653 10.1098/rspb.2002.2021 PMC1691045

[veaa019-B16] Earley E. J. et al (2009) ‘Inflorescences Contribute More than Rosettes to Lifetime Carbon Gain in *Arabidopsis thaliana* (Brassicaceae)’, American Journal of Botany, 96: 786–92. 21628233 10.3732/ajb.0800149

[veaa019-B17] Ebert D. et al (2004) ‘The Evolution of Virulence When Parasites Cause Host Castration and Gigantism’, The American Naturalist, 164: S19–32. 10.1086/424606 15540139

[veaa019-B18] Fornoni J. et al (2004) ‘Evolution of Mixed Strategies of Plant Defence Allocation against Natural Enemies’, Evolution, 58: 1685–95. 15446423 10.1111/j.0014-3820.2004.tb00454.x

[veaa019-B19] Fredensborg B. L., Poulin R. (2006) ‘Parasitism Shaping Host Life-History Evolution: Adaptive Responses in a Marine Gastropod to Infection by Trematodes’, Journal of Animal Ecology, 75: 44–53. 16903042 10.1111/j.1365-2656.2005.01021.x

[veaa019-B20] Fujisawa I. (1985) ‘Aphid Transmission of *Turnip mosaic virus* and *Cucumber mosaic virus*. 2. Transmission from Virus Mixtures’, Japanese Journal of Phytopathology, 51: 562–8.

[veaa019-B21] Gandon S., Agnew P., Michalakis Y. (2002) ‘Coevolution between Parasite Virulence and Host Life-History Traits’, The American Naturalist, 160: 374–88. 10.1086/341525 18707446

[veaa019-B22] Gioria M., Pyšek P., Osborne B. A. (2018) ‘Timing is Everything: Does Early and Late Germination Favor Invasions by Herbaceous Alien Plants?’, Journal of Plant Ecology, 11: 4–16.

[veaa019-B23] Goss E. M., Bergelson J. (2006) ‘Variation in Resistance and Virulence in the Interaction between *Arabidopsis thaliana* and a Bacterial Pathogen’, Evolution, 60: 1562–73. 17017057

[veaa019-B24] Hall S. R., Becker C., Caceres C. E. (2007) ‘Parasitic Castration: A Perspective from a Model of Dynamic Energy Budgets’, Integrative and Comparative Biology, 47: 295–309. 21672839 10.1093/icb/icm057

[veaa019-B25] Herms D. A., Mattson W. J. (1992) ‘The Dilemma of Plants: To Grow or Defend’, The Quarterly Review of Biology, 67: 283–335.

[veaa019-B26] Hily J. M. et al (2014) ‘The Relationship between Host Lifespan and Pathogen Reservoir Potential: An Analysis in the System *Arabidopsis thaliana*–*Cucumber mosaic virus*’, PLoS Pathogens, 10: e1004492. 25375140 10.1371/journal.ppat.1004492 PMC4223077

[veaa019-B27] Hily J. M. et al (2016) ‘Environment and Host Genotype Determine the Outcome of a Plant–Virus Interaction: From Antagonism to Mutualism’, New Phytologist, 209: 812–22. 26365599 10.1111/nph.13631

[veaa019-B28] Hochberg M. E., Michalakis Y., de Meeus T. (1992) ‘Parasitism as a Constraint on the Rate of Life-History Evolution’, Journal of Evolutionary Biology, 5: 491–504.

[veaa019-B29] Jones J. D. G., Dangl J. L. (2006) ‘The Plant Immune System’, Nature, 444: 323–9. 17108957 10.1038/nature05286

[veaa019-B30] Kover P. X., Schaal B. A. (2002) ‘Genetic Variation for Disease Resistance and Tolerance among *Arabidopsis thaliana* Accessions’, Proceedings of the National Academy of Sciences of the United State of America, 99: 11270–4. 10.1073/pnas.102288999 PMC123246 12172004

[veaa019-B31] Kutzer M. A., Armitage S. A. (2016) ‘Maximising Fitness in the Face of Parasites: A Review of Host Tolerance’, Zoology, 119: 281–9. 27373338 10.1016/j.zool.2016.05.011

[veaa019-B32] Kuznetsova A., Brockhoff P. B., Christensen R. (2017) ‘lmerTest Package: Tests in Linear Mixed Effects Models’, Journal of Statistic Software, 82: 1–26.

[veaa019-B33] Lafferty K. D., Kuris A. M. (2009) ‘Parasitic Castration: The Evolution and Ecology of Body Snatchers’, Trends in Parasitology, 25: 564–72. 19800291 10.1016/j.pt.2009.09.003

[veaa019-B34] Little T. J. et al (2010) ‘The Coevolution of Virulence: Tolerance in Perspective’, PLoS Pathogens, 6: e1001006. 20838464 10.1371/journal.ppat.1001006 PMC2936544

[veaa019-B35] López-González S. et al (2017) ‘An Infectious cDNA Clone of a Radish-Infecting *Turnip mosaic virus* Strain’, European Journal of Plant Pathology, 148: 207–11.

[veaa019-B36] Lunello P. et al (2007) ‘A Developmentally Linked, Dramatic, and Transient Loss of Virus from Roots *of Arabidopsis thaliana* Plants Infected by Either of Two RNA Viruses’, Molecular Plant-Microbe Interactions, 20: 1589–95. 17990966 10.1094/MPMI-20-12-1589

[veaa019-B37] Lynch M., Walsh B. 1998 Genetics and Analysis of Quantitative Traits. Sunderland, MA: Sinauer Associates, Inc.

[veaa019-B38] Manzano-Piedras E. et al (2014) ‘Deciphering the Adjustment between Environment and Life History in Annuals: Lessons from a Geographically–Explicit Approach in *Arabidopsis thaliana*’, PLoS One, 9: e87836. 24498381 10.1371/journal.pone.0087836 PMC3912251

[veaa019-B39] Mauricio R., Rausher M. D., Burdick D. S. (1997) ‘Variation in the Defence Strategies of Plants: Are Resistance and Tolerance Mutually Exclusive?’, Ecology, 78: 1301–11.

[veaa019-B40] McLeish M. et al (2019) ‘Co-Infection Organises Epidemiological Networks of Viruses and Hosts and Reveals Hubs of Transmission’, Phytopathology, 109: 1003–10. 30540552 10.1094/PHYTO-08-18-0293-R

[veaa019-B41] McLeod D. V., Day T. (2015) ‘Pathogen Evolution under Host Avoidance Plasticity’, Proceedings of the Royal Society B: Biological Sciences, 282: 20151656. 10.1098/rspb.2015.1656 PMC4571713 26336170

[veaa019-B42] Méndez-Vigo B. et al (2010) ‘Temporal Analysis of Natural Variation for the Rate of Leaf Production and Its Relationship with Flowering Initiation in *Arabidopsis thaliana*’, Journal of Experimental Botany, 61: 1611–23. 20190039 10.1093/jxb/erq032 PMC2852658

[veaa019-B43] Michalakis Y., Hochberg M. E. (1994) ‘Parasitic Effects on Host Life-History Traits: A Review of Recent Studies’, Parasite, 1: 291–4. 9140497 10.1051/parasite/1994014291

[veaa019-B44] Minchella D. J. (1985) ‘Host Life-History Variation in Response to Parasitism’, Parasitology, 90: 205–16.

[veaa019-B45] Montes N., Alonso-Blanco C., García-Arenal F. (2019) ‘*Cucumber mosaic virus* Infection as a Potential Selective Pressure on *Arabidopsis thaliana* Population’, PLoS Pathogens, 15: e1007810. 31136630 10.1371/journal.ppat.1007810 PMC6555541

[veaa019-B46] Montes N., Pagán I. (2019) ‘Light Intensity Modulates the Efficiency of Virus Seed Transmission through Modifications of Plant Tolerance’, Plants, 8: 304. 10.3390/plants8090304 PMC6783938 31461899

[veaa019-B47] Pagán I., Alonso-Blanco C., García-Arenal F. (2007) ‘The Relationship of within-Host Multiplication and Virulence in a *Plant*–Virus System’, PLoS One, 2: e786. 17726516 10.1371/journal.pone.0000786 PMC1950075

[veaa019-B48] Pagán I., Alonso-Blanco C., García-Arenal F. (2008) ‘Host Responses in Life-History Traits and Tolerance to Virus Infection in *Arabidopsis thaliana*’, PLoS Pathogens, 4: e1000124. 18704166 10.1371/journal.ppat.1000124 PMC2494869

[veaa019-B49] Pagán I., Alonso-Blanco C., García-Arenal F. (2009) ‘Differential Tolerance to Direct and Indirect Density–Dependent Costs of Viral Infection in *Arabidopsis thaliana*’, PLoS Pathogens, 5: e1000531. 19649316 10.1371/journal.ppat.1000531 PMC2712083

[veaa019-B50] Pagán I. et al (2010) ‘*Arabidopsis thaliana* as a Model for the Study of Plant-Virus Co-Evolution’, Philosophical Transactions of the Royal Society B: Biological Sciences, 365: 1983–95. 10.1098/rstb.2010.0062 PMC2880114 20478893

[veaa019-B51] Pagán I. et al (2014) ‘Vertical Transmission Selects for Reduced Virulence in a Plant Virus and for Increased Resistance in the Host’, PLoS Pathogens, 10: e1004293. 25077948 10.1371/journal.ppat.1004293 PMC4117603

[veaa019-B52] Pagán I., García-Arenal F., (2018) ‘Tolerance to Plant Pathogens: Theory and Experimental Evidence’, International Journal of Molecular Sciences, 19: 810. 10.3390/ijms19030810 PMC5877671 29534493

[veaa019-B53] Pinheiro J. et al (2018) *nlme: Linear and Nonlinear Mixed Effects Models*, R package version 3.1-137 <https://CRAN.R–project.org/package=nlme> accessed 6 Sep 2018].

[veaa019-B54] Poulin R., Morand S. (2000) ‘The Diversity of Parasites’, The Quarterly Review of Biology, 75: 277–93. 11008700 10.1086/393500

[veaa019-B55] R Core Team. (2018) R: A Language and Environment for Statistical Computing, R Foundation for Statistical Computing, Vienna, Austria.

[veaa019-B56] Råberg L. (2014) ‘How to Live with the Enemy: Understanding Tolerance to Parasites’, PLoS Biology, 12: e1001989. 25369060 10.1371/journal.pbio.1001989 PMC4219658

[veaa019-B57] Råberg L., Graham A. L., Read A. F. (2009) ‘Decomposing Health: Tolerance and Resistance to Parasites in Animals’, Philosophical Transactions of the Royal Society B: Biological Sciences, 364: 37–49. 10.1098/rstb.2008.0184 PMC2666700 18926971

[veaa019-B58] Rausher M. D. (2001) ‘Co-Evolution and Plant Resistance to Natural Enemies’, Nature, 411: 857–64. 11459070 10.1038/35081193

[veaa019-B59] Read A. F. (1994) ‘The Evolution of Virulence’, Trends in Microbiology, 2: 73–6. 8156274 10.1016/0966-842x(94)90537-1

[veaa019-B60] Restif O., Koella J. C. (2003) ‘Shared Control of Epidemiological Traits in a Coevolutionary Model of Host-Parasite Interactions’, The American Naturalist, 161: 827–36. 10.1086/375171 12858269

[veaa019-B61] Restif O., Koella J. C. (2004) ‘Concurrent Evolution of Resistance and Tolerance to Pathogens’, The American Naturalist, 164: E90–102. 10.1086/423713 15459887

[veaa019-B62] Rohr J. R., Raffel T. R., Hall C. A. (2010) ‘Developmental Variation in Resistance and Tolerance in a Multi-Host-Parasite System’, Functional Ecology, 24: 1110–21.

[veaa019-B64] Roy B. A., Kirchner J. W. (2000) ‘Evolutionary Dynamics of Pathogen Resistance and Tolerance’, Evolution, 54: 51–63. 10937183 10.1111/j.0014-3820.2000.tb00007.x

[veaa019-B65] Sánchez F. et al (1998) ‘Infectivity of *Turnip mosaic Potyvirus* cDNA Clones and Transcripts on the Systemic Host *Arabidopsis thaliana* and Local Lesion Hosts’, Virus Research, 55: 207–19. 9725673 10.1016/s0168-1702(98)00049-5

[veaa019-B66] Sánchez F. et al (2015) ‘Viral Strain–Specific Differential Alterations in Arabidopsis Developmental Patterns’, Molecular Plant-Microbe Interactions, 28: 1304–15. 26646245 10.1094/MPMI-05-15-0111-R

[veaa019-B67] Schlichting C., Pigliucci M. 1998 Phenotypic Plasticity: A Reaction Norm Perspective. Sunderland, MA: Sinauer Associates.

[veaa019-B68] Sharbel T. F., Haubold B., Mitchell-Olds T. (2000) ‘Genetic Isolation by Distance in *Arabidopsis thaliana*: Biogeography and Postglacial Colonization of Europe’, Molecular Ecology, 9: 2109–18. 11123622 10.1046/j.1365-294x.2000.01122.x

[veaa019-B69] Shuckla A., Pagán I., García-Arenal F. (2018) ‘Effective Tolerance Based on Resource Reallocation is a Virus-Specific Defence in *Arabidopsis thaliana*’, Molecular Plant Pathology, 19: 1454–65. 29027740 10.1111/mpp.12629 PMC6638070

[veaa019-B70] Simms E., Triplett J. (1994) ‘Costs and Benefits of Plant Responses to Disease: Resistance and Tolerance’, Evolution, 48: 1973–85. 28565152 10.1111/j.1558-5646.1994.tb02227.x

[veaa019-B71] Sokal R. R., Rohlf F. J. (1995) Biometry: The Principles and Practices of Statistics in Biological Research. New York, NY: WH Freeman.

[veaa019-B72] Stearns S. C. (1992) The Evolution of Life Histories. London, UK: Oxford University Press.

[veaa019-B73] Strauss S. Y., Agrawal A. A. (1999) ‘The Ecology and Evolution of Plant Tolerance to Herbivory’, Trends in Ecology & Evolution, 14: 179–85. 10322530 10.1016/s0169-5347(98)01576-6

[veaa019-B74] Syller J. (2012) ‘Facilitative and Antagonistic Interactions between Plant Viruses in Mixed Infections’, Molecular Plant Pathology, 13: 204–16. 21726401 10.1111/j.1364-3703.2011.00734.x PMC6638836

[veaa019-B75] Thompson K., Stewart A. (1981) ‘The Measurement and Meaning of Reproductive Effort in Plants’, The American Naturalist, 117: 205–11.

[veaa019-B76] Vale P. F., Little T. J. (2012) ‘Fecundity Compensation and Tolerance to a Sterilizing Pathogen in Daphnia’, Journal of Evolutionary Biology, 25: 1888–96. 22856460 10.1111/j.1420-9101.2012.02579.x PMC3798115

[veaa019-B77] van der Meijden E., Wijn H., Verkaar J. (1988) ‘Defence and Regrowth: Alternative Plant Strategies in the Struggle against Herbivores’, Oikos, 51: 355–63.

[veaa019-B78] Vijayan V. et al (2017) ‘Virulence Evolution of a Sterilizing Plant Virus: Tuning Multiplication and Resource Exploitation’, Virus Evolution, 3: vex033. 29250431 10.1093/ve/vex033 PMC5724401

[veaa019-B79] Woolhouse M. E. J. et al (2002) ‘Biological and Biomedical Implications of the Co-Evolution of Pathogens and Their Hosts’, Nature Genetics, 32: 569–77. 12457190 10.1038/ng1202-569

[veaa019-B1168838] Zhang L., Hanada K., Palukaitis P. (1994) ‘Mapping Local and Systemic Symptom Determinants of Cucumber Mosaic Cucumovirus in Tobacco’, Journal of General Virology, 75: 3185–91. 7964627 10.1099/0022-1317-75-11-3185

