# Supplementary material for: Trade-offs between host tolerances to different pathogens in plant–virus interactions
Source: Virus Evol. 2020 Mar 18;6(1):veaa019. doi: 10.1093/ve/veaa019 (PMC7079720; doi:10.1093/ve/veaa019)
Supplement: veaa019_Supplementary_Data [file veaa019_supplementary_data.zip › Supp/Montesetal.Suppl_figure_Virus_Evol.Final.pdf]

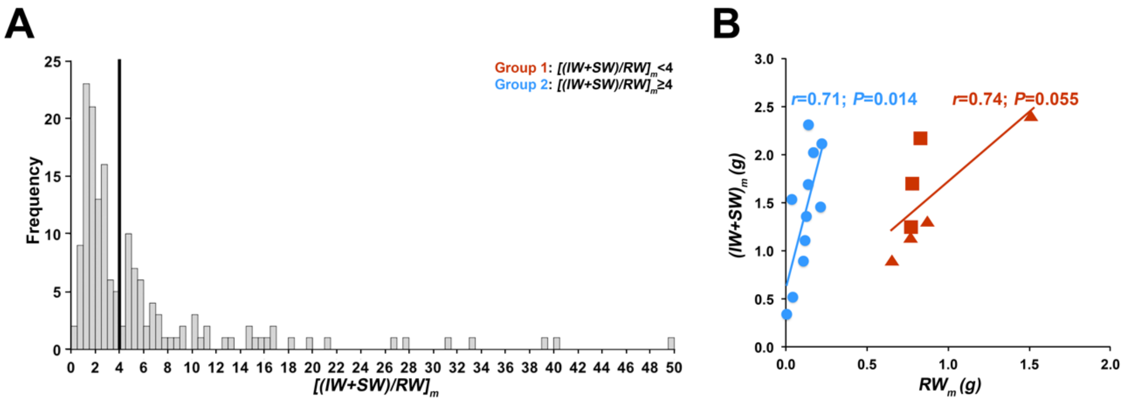

Figure S1. Relationship between rosette growth (*RW*) and reproduction (inflorescence plus seeds weights, *IW*+*SW*) in Arabidopsis genotypes. Panel A: Frequency distribution of the (*IW*+*SW*)/*RW* relationship of the 18 genotypes, based on individual plant values. Vertical black bar indicates the threshold value defining the two allometric groups. Panel B: Correlation between *IW*+*SW* and *RW* for allometric Group 1 (red) and Group 2 (blue) using mean plant genotype values of mock-inoculated (m) plants. Values for plant genotypes of subgroups 1a (rectangle) and 1b (triangle) are also shown. Data are mean ± standard error of *RW* and *IW*+*SW* in g of mock inoculated plants.
